# A water extract of *Malva verticillata* seeds suppresses osteoclastogenesis and bone resorption stimulated by RANK ligand

**DOI:** 10.1186/s12906-016-1295-6

**Published:** 2016-08-31

**Authors:** Ki-Shuk Shim, Chung-Jo Lee, Nam-Hui Yim, Hyunil Ha, Jin Yeul Ma

**Affiliations:** KM Application Center, Korea Institute of Oriental Medicine, 70 Cheomdanro Dong-gu, Daegu, 41062 Republic of Korea

**Keywords:** Malvaceae, *Malva verticillata* seeds, Receptor activator of nuclear factor-kB ligand, Osteoclasts, Nuclear factor of activated T-cells, cytoplasmic 1

## Abstract

**Background:**

*Malva verticillata* seeds are used as a therapeutic medicine to treat kidney dysfunction in traditional Chinese medicine (TCM). TCM has suggested that herbal medicine tonifying kidney function may have beneficial effect on bone metabolism.

**Methods:**

Osteoclastogenesis was examined in bone marrow macrophages by measuring tartrate-resistant acid phosphatase (TRAP) activity and counting the number of TRAP-stained multinuclear cells. The activation of receptor activator of nuclear factor-kB (RANK) ligand signaling, and the expression of c-Fos and nuclear factor of activated T-cells, cytoplasmic 1 (NFATc1) were investigated by western blot analysis. Transcription factor and bone resorption marker mRNA levels were evaluated using real-time quantitative polymerase chain reaction. The bone resorption activity of mature osteoclast was examined in osteoclasts cultured on a hydroxyapatite-coated culture plate.

**Results:**

A water extract of *M. verticillata* seeds (WEMV) inhibited osteoclastogenesis stimulated by RANKL. WEMV also strongly inhibited expression of c-Fos and NFATc1 as well as phosphorylation of c-Jun N-terminal kinase, p38, I-kBα, and phospholipase γ2. Furthermore, WEMV significantly attenuated osteoclast resorption activity and downregulated mRNA expression of resorption markers.

**Conclusion:**

These results demonstrate that WEMV inhibits osteoclastogenesis and bone resorption by suppressing the RANKL signaling pathway and suggest that *M. verticillata* seeds may be used as a therapeutic candidate in complementary alternative medicine to treat pathological bone diseases.

## Background

Osteoporosis is a skeletal disorder characterized by low bone mineral density and deteriorated bone microstructure, which increases the incidence of bone fracture. It is caused mainly by an imbalance between bone resorption and bone formation when an increased rate of bone resorption overcomes a decreased rate of bone formation caused by estrogen deficiency, aging, or an inadequate intake of calcium or vitamin D3, especially in elderly populations [1, 2]. Receptor activator for nuclear factor-kB (RANK) ligand (RANKL) is a cytokine that is essential for osteoclast differentiation, regulation of osteoclast bone resorbing activity and osteoclast apoptosis [3]. RANKL activates various signaling molecules and critical transcription factors in osteoclastogenesis such as mitogen-activated protein kinase (MAPK) and nuclear factor of activated T-cells, cytoplasmic 1 (NFATc1). Recent studies have investigated the molecular mechanism of osteoclastogenesis and bone resorption to develop anti-osteoporosis agents with fewer undesirable side effects [4, 5]. Herbal medicine and natural products have recently been suggested to mitigate bone loss and ameliorate osteoporosis with fewer side effects through their ability to affect bone cells [6–8].

*Malva verticillata* Linn is a species of the mallow genus *Malva* in the family of Malvaceae [9]. Seeds of *M. verticillata*, commonly known as Dong Kui Zi or Donggyuja, have been used to enhance kidney strength, remove renal stones, and treat chronic mastitis and hypogalactia in traditional Chinese medicine (TCM) [9, 10]. *M. verticillata* seeds contain various polysaccharides and flavonoids with a number of pharmacological activities including hypoglycemic activity [11–13]. In TCM, herbal medicine is used to invigorate kidney function so as to strengthen musculoskeletal organs such as bone [14, 15]. Chinese herbal formulas or herbal medicines prescribed in TCM to nourish or replenish kidney function have been shown to have a positive effect on ovariectomy-induced osteoporosis [16] and a pharmaceutical effect on bone by regulating osteoclast differentiation [17] or mast cell activity [18]. Based on the results of these studies, *M. verticillata* seeds used in TCM to stimulate kidney function might have a beneficial effect on bone by regulating bone cell differentiation; however, no study has explored the effect of *M. verticillata* seeds on bone cell differentiation and/or function.

In this study, we examined whether a water extract of *M. verticillata* seeds (WEMV) exerts a pharmaceutical effect to regulate osteoclastogenesis or osteogenesis. We found that WEMV inhibited RANKL-induced osteoclastogenesis, but WEMV had no effect on bone morphogenetic protein-2 (BMP-2)- or ascorbic acid/β-glycerophosphate-induced osteogenesis. To increase our understanding of the molecular mechanism underlying WEMV activity in osteoclastogenesis and bone resorption, we investigated the effect of WEMV on RANKL signaling and the expression of genes involved in bone resorption.

## Methods

### Preparation of a water extract of *M. verticillata* seeds

*M. verticillata* seeds (Yeongcheon herb, Korea) were authenticated by Prof. K.H. Bae (Chungnam National University, Korea). A voucher specimen with registration number 221 is maintained in the herbarium of the KM Application Center at the Korea Institute of Oriental Medicine. Dried *M. verticillata* seeds (50 g) were extracted by boiling in distilled water (1 L) for 3 h as described previously [19]. The water extract of *M. verticillata* seeds (WEMV) was filtrated using standard sieves (150 μm), concentrated by lyophilization, and stored at –20 °C until use. A WEMV stock solution was prepared by dissolving the lyophilized powder in distilled water followed by filtering through a 0.2 μm syringe filter. The yield of dried extract from the staring materials was approximately 8.96 % (w/w).

### Gas chromatography/ mass spectrometry analysis

The lyophilized water extract of *M. verticillata* seeds was submitted to separation through gas chromatography with detection by mass spectrometry (GC/MS). The analysis was performed in a GC/MS system (Agilent Technologies, Atlanta, GA, USA) using DB-5 MS capillary column (30 m × 0.25 mm × 0.25 μm). Chromatographic conditions were as follows: the extract (1 μL) was injected in split mode with a ratio of 1/20 at 350 °C, oven initial temperature was 70 °C during 1 min, followed by heating at a rate of 5 °C/min at 300 °C, and totally run for 67 min. The mass analyzer was set to scan from 10 to 800 atomic mass unit. β-sitosterol was used as standard marker for GC/MS analysis. Peak identification was carried out comparison of the experimental mass spectrum in the National Institute of Standards and Technology (NIST) and Wiley GC-MS libraries. Fourteen components, including 1,3-dihydroxyacetone dimer, d-alanine, 5-hydroxymethyl furfural, 2-hydroxy-gamma-butyrolactone, palmitic acid, oleamide, and β-sitosterol, were identified in WEMV (data not shown).

### Cell culture

Mouse bone marrow macrophages (BMMs) were obtained from mouse bone marrow cells (BMCs) as described previously [20]. For osteoclast differentiation, BMMs (1 × 10^4^ cells/well, 96-well plate) were cultured in α-MEM differentiation medium containing 60 ng/mL of macrophage colony stimulating factor (M-CSF) and 100 ng/mL of RANKL for 4 days. Tartrate-resistant acid phosphatase (TRAP)-positive stained cells with more than three nuclei were counted as multinuclear osteoclasts [TRAP (+) MNCs]. For osteoblast differentiation, C2C12 cells (ATCC, USA) were cultured (1 × 10^4^ cells/well, 96-well plate) in α-MEM differentiation medium containing 100 ng/mL of BMP-2 for 3 days. Primary calvarial osteoblasts (5 × 10^4^ cells/well, 48-well plate) obtained as described previously [21] were cultured in α-MEM differentiation medium containing 50 μg/mL ascorbic acid and 10 mM β-glycerophosphate for 5 days. BMMs were cultured in α-MEM proliferation medium, and C2C12 cells and osteoblasts were cultured in DMEM proliferation medium containing various concentrations of WEMV for 2 days to examine cytotoxicity using a CCK-8 assay (Dojindo Molecular Technologies Inc., Tokyo, Japan). All media contained 10 % fetal bovine serum (FBS) and antibiotics (100 U/mL penicillin and 100 μg/mL streptomycin) and were replaced at 3-days intervals.

### Enzyme assay

TRAP and alkaline phosphatase (ALP) activity assay were performed as described previously [20]. Briefly, cells were fixed in 10 % formalin, permeabilized with 0.1 % Triton X-100, and incubated with TRAP assay buffer (50 mM sodium tartrate, 0.12 M sodium acetate, pH 5.2) with *p*-nitrophenyl phosphate (1 mg/mL) or incubated with ALP assay buffer [100 mM sodium carbonate (pH 10), 1 mM MgCl_2_, 50 mM Na_2_CO_3_] with naphthol AS-BI phosphate. After a 10-min incubation at 37 °C, the reaction was stopped with stop solution and the absorbance was measured. MNCs were stained with TRAP staining buffer containing naphthol AS-MX phosphate and Fast Red Violet LB (Sigma-Aldrich).

### Western blot analysis

Cells were washed twice with phosphate-buffered saline (PBS) and total cell lysate was obtained using Radioimmunoprecipitation assay (RIPA) buffer (Millipore, MA, USA) containing protease and phosphatase inhibitors. Protein concentration was determined using a bicinchoninic acid assay (BCA) Assay Kit (Thermo Scientific, Rockford, IL, USA). Total protein (30 μg) was separated by 12.5 % SDS-PAGE gel electrophoresis, transferred to polyvinylidene fluoride (PVDF) membrane, and immunoblotted with specific antibody. Antibodies for MAPK, nuclear factor-kappaB (NF-kB), or phospholipase C (PLC) signaling proteins (Cell Singling Technology, MA, USA) or c-Fos and NFATc1 transcription factors (Santa Cruz Biotechnology, CA, USA) were used in this study. Chemiluminescent signals were detected using a ChemiDoc imaging system (Bio-Rad Laboratories, CA, USA) and a chemiluminescence reagent (Thermo Scientific).

### Real-time quantitative polymerase chain reaction (qRT-PCR)

Total RNA was isolated using an RNAspin total RNA extraction kit (Intron, Daejeon, Korea). cDNA was synthesized from 1 μg of total RNA using AccuPower RT-PreMix (Bioneer, Daejeon, Korea) according to the manufacturer’s protocol. qRT-PCR analysis was performed using a CFX96 Touch Real-Time PCR System (Bio-Rad, CA, USA) and AccuPower GreenStar qPCR Master Mix (Bioneer, Daejeon, Korea). The qRT-PCR reactions comprised 40 cycles at 94 °C for 20 s and 60 °C for 40 s. All reactions were performed in triplicate. The relative expression levels of target genes were normalized to hypoxanthine phosphoribosyltransferase (HPRT) expression and analyzed using Bio-Rad CFX Manage 3.1 software (Bio-Rad, CA, USA).

### Retrovirus preparation and infection

A pMX-puro-green fluorescent protein (GFP) retrovirus vector (provided by Dr. T. Kitamura, University of Tokyo) and a pMX-Ca-NFATc1 vector encoding HA-tagged Ca-NFATc1, a constitutively expressed form of NFATc1 provided by Dr. N.A. Clipstone (Northwestern University), were used for retrovirus preparation as described previously [20]. Briefly, to generate retroviral stocks, the vector was transfected into Plat-E retroviral packaging cells. BMMs (3 × 10^6^ cells, 100-mm dish) were infected with retrovirus soup from the packaging cells by incubation with polybrene (6 μg/mL) for 8 h. Infected BMMs were selected with puromycin (2 μg/mL, Sigma-Aldrich) for 5 days. The selected BMMs were cultured in differentiation medium with WEMV for 4 days and then stained with TRAP-staining solution.

### Bone resorption assay

Osteoclasts obtained from a BMCs-osteoblasts coculture system were cultured on OsteoAssay Surface plates (Corning, MA, USA). Osteoclasts were preincubated with WEMV for 3 h and further cultured in differentiation medium for an additional 16 h. After removing cells, stained osteoclasts and resorbed areas were observed under an inverted microscope (40× magnification). The resorption areas were analyzed in three randomly selected fields of each well using Image J software.

### Statistical analysis

Statistical significance of the differences in TRAP activity, MNC number, and mRNA levels of genes was analyzed using Student’s *t*-test. Data are presented as means ± the standard deviation of three independent experiments. Differences are considered significant at *p* < 0.05.

## Results

### WEMV inhibits RANKL-induced osteoclast differentiation in BMMs

**Fig. 1:**
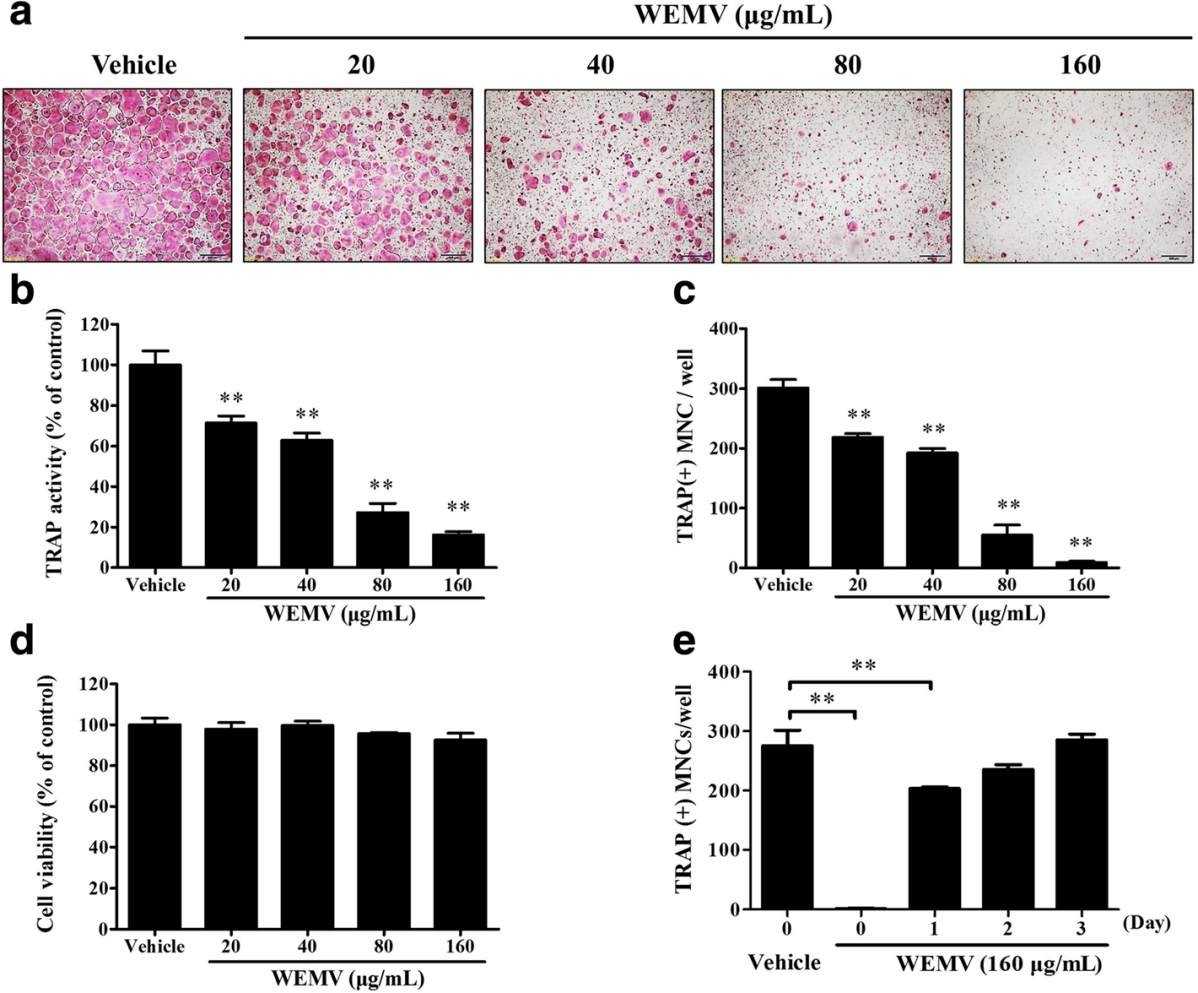
WEMV has a negative effect on osteoclastogenesis. BMMs were cultured for 4 days in differentiation medium containing WEMV at concentrations of 20, 40, 80, and 160 μg/mL. **a** TRAP-positive stained cells were visualized using TRAP staining solution. **b** TRAP activity was measured by TRAP assay with *p*-nitrophenyl phosphate as described in the Methods. **c** TRAP-positive stained MNCs were counted under an inverted microscope (40× magnification). **d** Cell viability percentage were determined using a CCK assay after a 2-day incubation with the indicated concentrations of WEMV. **e** BMMs were cultured in differentiation medium with WEMV (160 μg/mL) for the indicated numbers of days. TRAP-positive stained MNCs were counted on day 4. **p < 0.01 versus control

We first examined the pharmaceutical effect of WEMV on osteoclast differentiation. BMMs were cultured in differentiation medium with or without WEMV for 4 days. As shown in Fig. 1a, RANKL induced differentiation of BMMs into TRAP-positive stained MNCs; however, WEMV inhibited RANKL-induced TRAP activity and MNC formation (*p* < 0.01) (Fig. 1b and c) in a dose-dependent manner. WEMV at a concentration of 160 μg/mL almost completely suppressed the MNC number (9 ± 2, *p* < 0.01) without inducing cytotoxicity compared with the control (301 ± 11) (Fig. 1d) suggesting that inhibitory activity of WEMV was specific for osteoclastogenesis. However, because osteoclastogenesis involves sequential steps in the differentiation of precursor cells into pre-osteoclast and osteoclasts, the stage of osteoclastogenesis that was inhibited by WEMV was unclear. To determine the effect of WEMV on each stage of osteoclastogenesis, BMMs were cultured with WEMV (160 μg/mL) at four time points after which MNC numbers were counted on day 4. We found that the MNC number was suppressed significantly when WEMV was added on day 0 (2 ± 1, *p* < 0.01) or on day 1 (203 ± 2, *p* < 0.01) as compared with the control (275 ± 22) (Fig. 1e), but MNC formation was not inhibited by WEMV when added on days 2 or 3 suggesting that WEMV inhibits early osteoclast differentiation.

### WEMV does not affect osteoblast differentiation

**Fig. 2:**
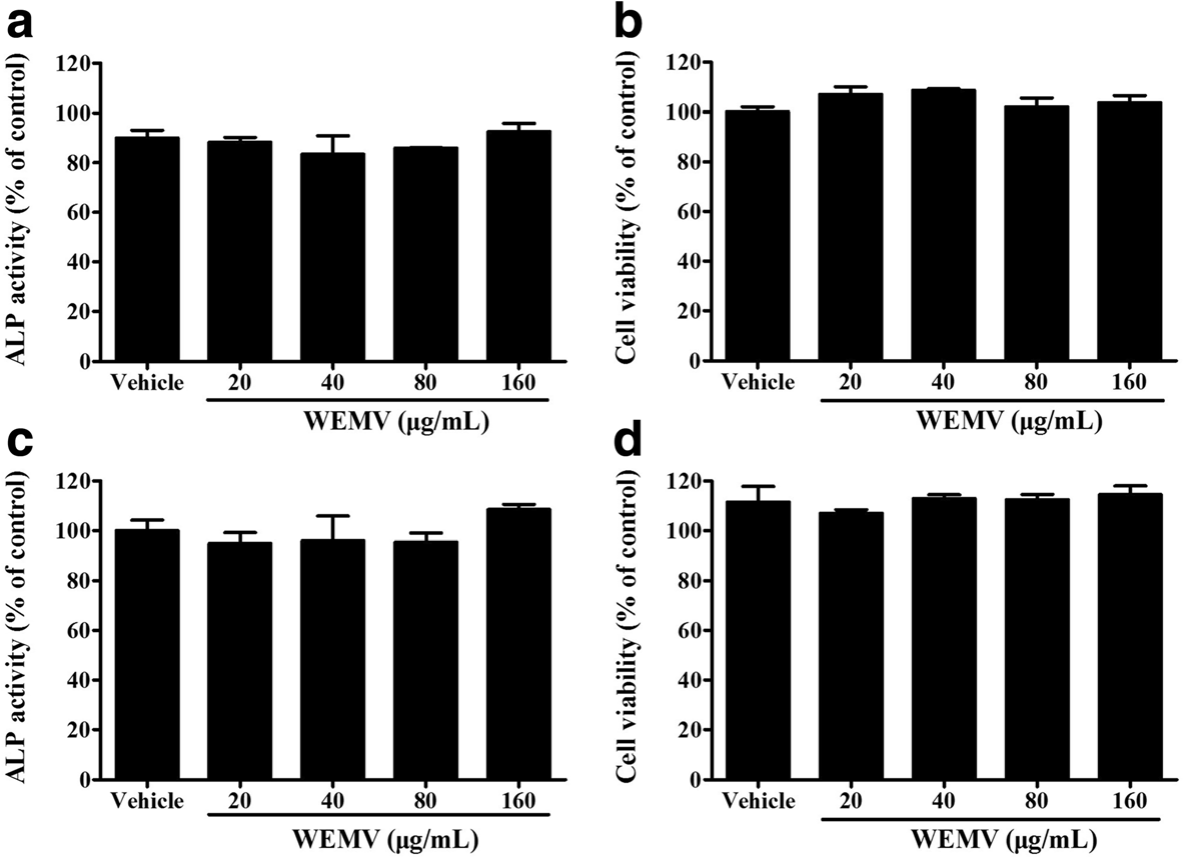
WEMV does not affect osteoblast differentiation. C2C12 cells were cultured in differentiation medium with the indicated concentrations of WEMV for 3 days. **a** ALP activity of C2C12 cells was determined using an ALP assay with naphthol AS-BI phosphate as described in the Methods. **b** A CCK-8 assay was performed to determine the viability of C2C12 cells after a 2-day incubation with the indicated concentrations of WEMV. Calvarial osteoblast cells were cultured in differentiation medium for 15 days. **c** ALP activity and (**d**) calvarial osteoblast cell viability were examined after 15- and 2-day incubations, respectively

BMP-2 and ascorbic acid/β-glycerophosphate induce osteoblast differentiation accompanied by an increase in differentiation markers such as ALP. We examined whether WEMV has any effect on osteoblast differentiation of C2C12 or calvarial osteoblast cells by measuring ALP activity. We found that WEMV did not increase BMP-2-induced ALP activity or decrease the viability of C2C12 cells, even at high concentration (160 μg/mL) (Fig. 2a and b). We also found that WEMV did not affect osteoblast differentiation or viability of calvarial cells (Fig. 2c and d). These results suggested that WEMV does not regulate the early stage of osteoblast differentiation and proliferation.

### WEMV suppresses NFATc1 and c-Fos expression in BMMs

**Fig. 3:**
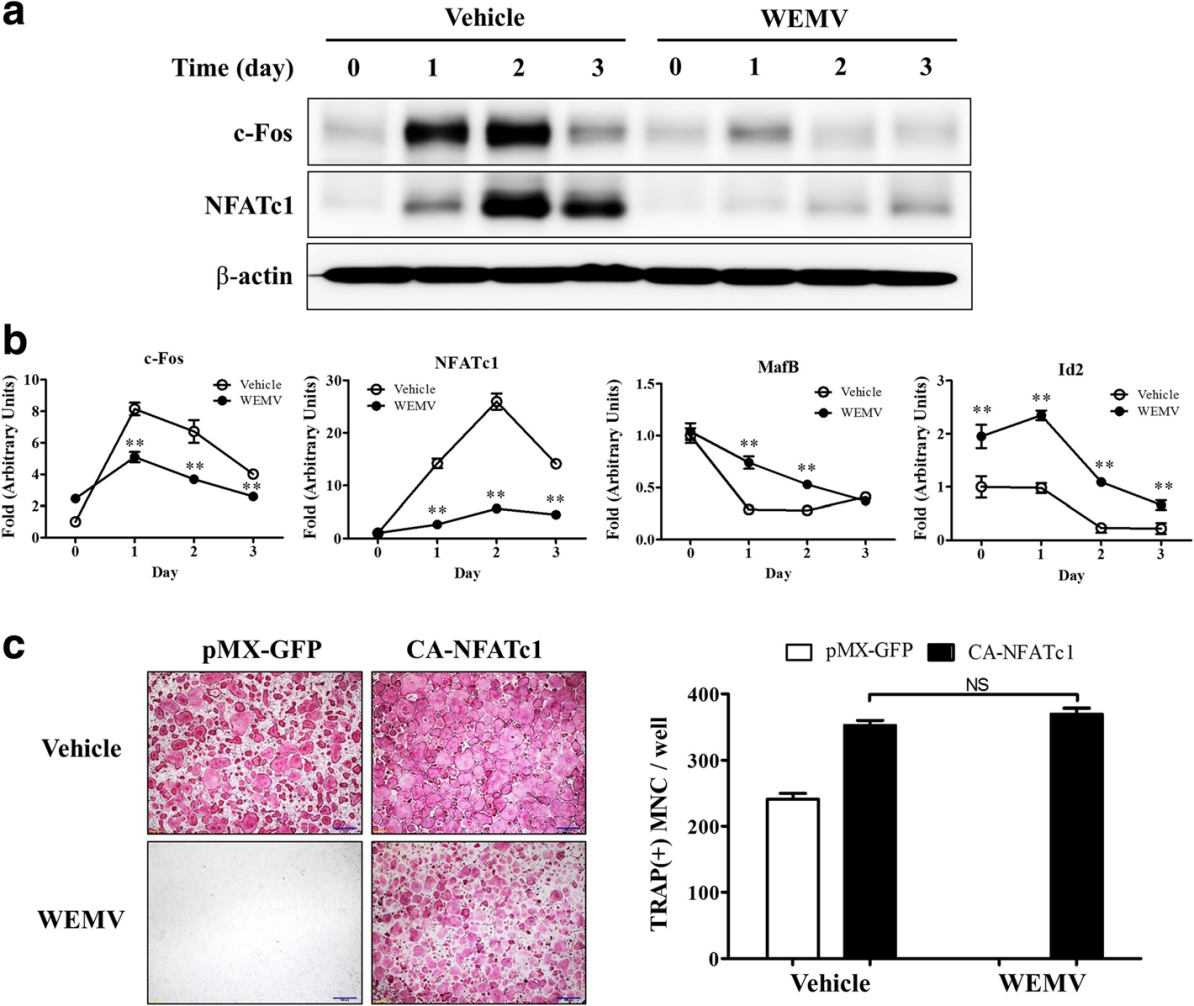
WEMV downregulates c-Fos and NFATc1 expression. BMMs were pretreated with WEMV (160 μg/mL) for 3 h and cultured in differentiation medium for the indicated times as described in the Methods. Total RNA or protein was obtained at the indicated time points. **a** Total cell lysates (30 μg) were separated by SDS-PAGE electrophoresis and transferred to PVDF membranes. Western blot analysis was performed with antibodies specific for c-Fos (top), NFATc1 (middle), and β-actin (bottom). **b** qRT-PCR analysis of c-Fos, NFATc1, MafB, and Id2 was performed at the indicated time points. Each mRNA level was normalized to that of HPRT. **c** BMMs were infected with retrovirus vector harboring GFP (control) or CA-NFATc1 in the presence of polybrene (6 μg/mL) for 8 h. The infected cells were cultured in α-MEM medium containing puromycin (2 μg/mL) for 5 days and then cultured in differentiation medium containing WEMV (160 μg/mL) for 4 days. TRAP-positive stained MNCs were counted after TRAP staining under an inverted microscope (40× magnification). **p < 0.01 versus control. NS; not significance

To determine how WEMV suppresses osteoclastogenesis, we investigated c-Fos and NFATc1 expression. BMMs pretreated with WEMV and control were further incubated in osteoclast differentiation medium for various time periods. c-Fos and NFATc1 mRNA and protein levels increased markedly on days 1 and 2 in response to RANKL stimulation (Fig. 3a and b), but WEMV almost completely abolished their protein levels. WEMV reduced c-Fos mRNA level to 63 % on day 1 and NFATc1 mRNA level to 22 % on day 2 (*p* < 0.01) relative to control. Since NFATc1 is the master transcription factor that regulates osteoclastogenesis, we investigated whether ectopic NFATc1 expression could restore osteoclastogenesis that was suppressed by WEMV. BMMs were infected with control retroviral vector or a recombinant retroviral vector harboring a CA-NFATc1 and were then cultured under osteoclast differentiation conditions. WEMV completely inhibited MNC formation in BMMs infected with the control vector, but did not inhibit MNC formation in BMMs infected with CA-NFATc1 (Fig. 3c). As a counterpoint to the effect of WEMV on NFATc1-inducing factors such as c-Fos, we investigated the mRNA expression of v-Maf avian musculoaponeurotic fibrosarcoma oncogene homolog B (MafB) and inhibitor of DNA binding 2 (Id2), which are transcriptional repressors of NFATc1 in BMMs. WEMV significantly attenuated RANKL-induced downregulation of MafB mRNA level to 76 % and increased Id2 mRNA level by two fold on day 1 (*p* < 0.01) relative to the control (Fig. 3b). These results indicated that the effect of WEMV on osteoclastogenesis may involve the inhibition of NFATc1 expression.

### WEMV inhibits MAPK, NF-kB, and PLCγ2 activation in BMMs

**Fig. 4:**
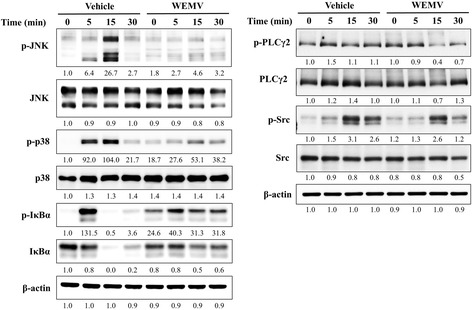
WEMV suppresses MAPK, NF-kB, and PLCγ signaling pathways. BMMs pretreated with WEMV (160 μg/mL) for 3 h were exposed to RANKL for the indicated times. Total cell lysate (30 μg/mL) were separated by SDS-PAGE electrophoresis, transferred to PVDF membrane, and immunoblotted with antibodies specific for each signaling pathway

RANKL-RANK interaction activates early signaling pathways, including MAPK and NF-kB signaling, to regulate NFATc1 expression during osteoclastogenesis. We further explored WEMV inhibition of early-stage of osteoclast differentiation by examining the effect of WEMV on RANKL-induced activation of MAPK and NF-kB signaling. As shown in Fig. 4, RANKL increased c-Jun N-terminal kinase (JNK) and p38 phosphorylation markedly (lane 3) as well as I-kBα phosphorylation (lane 2) and degradation (lane 3). However, WEMV decreased JNK phosphorylation (lane 7) and p38 phosphorylation (lane 7) at 15 min relative to the control. In addition, WEMV partially reduced I-kBα phosphorylation (lane 6) at 5 min and impaired I-kBα degradation (lane 7 and 8) at 15 and 30 min relative to the control. We next evaluated whether WEMV affects Src-PLC-Ca^2+^ pathway to regulate NFATc1 expression. WEMV markedly suppressed RANKL-induced PLCγ2 phosphorylation (lane 6) at 5 min and Src phosphorylation (lane 7) at 15 min.

### WEMV inhibits bone resorption activity of osteoclasts

**Fig. 5:**
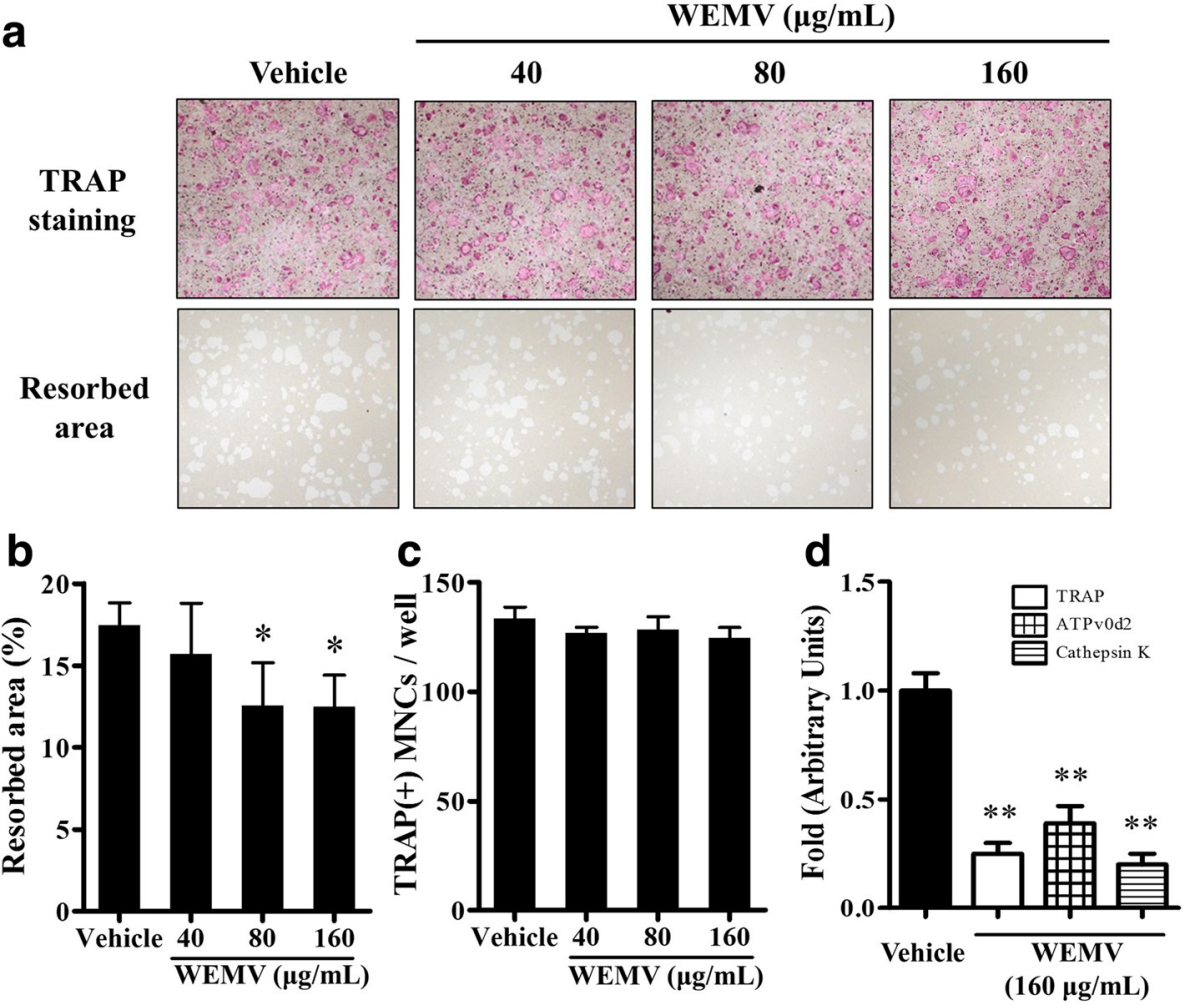
WEMV inhibits bone resorption. Osteoclasts with bone resorbing function were obtained from coculture of BMCs and calvarial osteoblasts. The cells were seeded on an OsteoAssay Surface plate and cultured in differentiation medium containing WEMV (160 μg/mL) for 16 h. **a** MNCs (upper panel) or the resorbed areas (lower panel) were photographed under an inverted microscope (40× magnification). **b** The percentage of resorbing areas on the plate was quantified using Image J software. **c** TRAP-positive stained MNCs were counted to evaluate the cytotoxic effect of WEMV on mature osteoclasts. **d** The mRNA levels of bone resorption genes (TRAP, ATPv0d2, and cathepsin K) were analyzed by qRT-PCR. *p < 0.05; **p < 0.01 versus control

To examine WEMV activity on bone resorption in a physiologically relevant manner, mature osteoclasts obtained from a BMCs-osteoblasts coculture system were cultured with or without WEMV on a hydroxyapatite-coated plate. As shown in Fig. 5a, RANKL treatment stimulated mature osteoclasts to resorb on the plate. However, WEMV substantially decreased the resorption area without affecting the MNC number (Fig. 5b and c). Quantitative analysis of the resorption area demonstrated that WEMV concentrations of 80 μg/mL and 160 μg/mL significantly suppressed bone resorption activity of osteoclasts to 12.56 ± 1.86 and 12.49 ± 1.37 % (*p* < 0.05), respectively, relative to the control (17.46 ± 1.07 %). To delineate the molecular mechanism underlying the effect of WEMV on bone resorption, we examined whether WEMV affects the expression of TRAP, ATPv0d2, and cathepsin K, all of which are involved in bone resorption. We found that WEMV caused marked downregulation of the mRNA levels of TRAP, ATP6v0d2, and cathepsin K to 25, 39, and 20 % (*p* < 0.01), respectively, relative to the control (Fig. 5d).

## Discussion

*M. verticillata* seeds have been used in Asia for the treatment of nephrolithiasis and cystolithiasis and for enhancing kidney strength and reducing lower back and bladder pain. The aim of this study was to evaluate the pharmaceutical effect of WEMV on osteoclastogenesis and osteogenesis. We showed that WEMV inhibited osteoclastogenesis by decreasing NFATc1 expression and inhibited bone resorption by downregulating the expression of resorption markers, albeit at high concentration.

We found that WEMV suppressed osteoclast differentiation (Fig. 1) and NFATc1 expression significantly (Fig. 4). NFATc1 plays a crucial role in the regulation of osteoclast transcription during osteoclastogenesis. Upon RANKL stimulation, initiation and/or amplification of NFATc1 expression is achieved mainly by the activation of MAPK, NF-kB, and PLCγ-activated Ca^2+^ signaling pathways [22] which subsequently activate key transcription factors, activator protein 1 (AP-1), NFATc1, and cAMP response element binding (CREB) [3]. These RANKL-stimulated pathways are somewhat sequential with respect to NFATc1 induction, but they cooperate in the amplification of NFATc1 expression to regulate and facilitate osteoclast-specific transcription. Our results showed that WEMV inhibited the activation of MAPK, NF-kB, and PLCγ signaling as well as suppressed c-Fos expression significantly (Figs. 3 and 4), suggesting that WEMV inhibition to the upstream signaling cascade of NFATc1 suppresses NFATc1 expression and osteoclastogenesis. Regarding distinct activation of these signaling pathways for NFATc1 expression, different inhibitory components present in WEMV might affect each signaling pathway during osteoclastogenesis.

WEMV attenuated RANKL-induced downregulation of MafB and Id2 significantly at days 1 and 2 accompanied by suppression of NFATc1 expression (Fig. 3). Transcription repressors, such as MafB and Id2, are expressed abundantly in BMMs to interrupt the formation of transcriptional complex or the ability of complexes to transcriptionally regulate NFATc1 expression thereby preventing non-specific initiation of osteoclastogenesis and maintaining the potential of BMMs to differentiate [23]. In response to RANKL or TNFα stimulation, the expression of transcription repressors is downregulated markedly within 48 h; however, their ectopic expression blocks osteoclastogenesis [24, 25]. Thus, WEMV activity to maintain the expression of transcription repressor may partially contribute to suppress NFATc1 expression and thus block osteoclastogenesis, although RANKL stimulates osteoclast precursors.

Bone resorption requires high levels of resorption markers such as TRAP, ATPv0d2, and cathepsin K, in mature osteoclasts to degrade bone matrix. Inhibition of bone resorption markers such as cathepsin K results in decreased resorption activity by disrupting the intracellular vesicle transport system that delivers degraded collagen [26]. Our results showed that WEMV suppressed either bone resorption or transcription of all of the above marker genes significantly in osteoclasts without affecting the MNC number (Fig. 5), suggesting that WEMV may block either generation of resorbing protein or vesicular trafficking system for bone resorption. With respect to the inhibition of bone resorption and osteoclastogenesis by WEMV with no effect on osteoblast differentiation or proliferation, an evaluation of the anti-osteoporosis potential of WEMV alone or in combination with TCM stimulating osteogenesis in an *in vivo* animal model system representing pathological bone disorders would be of interest for a future study.

*M. verticillata* seeds contains several chemical constituents, such as polysaccharides, fatty acid, flavonoid, terpenes, and sterol [10]. Different polysaccharides, such as MVS-I, MVS-IIA, or MVS-V, has been identified as major polysaccharide in water extract of *M. verticillata* seeds [27, 28]. Among them, arabinogalactan, a part component of MVS-I or MVS-IIA, weakly inhibits osteoclast differentiation of RAW264.7 cells (13.8 % inhibition at 100 μg/ml) by downregulating the expression of TRAP and cathepsin K [29]. However, peptidoglycan, other polysaccharides identified in water extract of *M. verticillata* seeds, is known to have stimulatory effect on osteoclast differentiation as well as bone resorption [30], which should be avoided for anti-osteoclast agents. Besides polysaccharide, *M. verticillata* seeds also have oil constituents (11 % of total yield) including 16.18 % palmitic, 7.7 % oleic, 61.6 %, linoleic, and 7.9 % stearic acids [10]. We also identified the presence of fatty acid (palmitic acid and oleamide) and sterol (β-sitosterol) in WEMV by GC/MS analysis (data not shown). Regarding the inhibitory activity of fatty acid and sterol on osteoclast differentiation, palmitic acid (50 % inhibition at 10 μg/ml) inhibits osteoclastogenesis by suppressing the expression of fatty acid binding receptor, while oleamide (150 % inhibition at 100 μM) suppresses bone resorption by inhibiting gap junction of osteoclasts [31, 32]. In addition, several terpenes and sterol (β-sitosterol; 15 % inhibition at 1 μM) inhibit osteoclast differentiation by inhibiting TRAP activity although the possible action mechanism is unknown [33]. Thus, considering the inhibitory activity of WEMV at high concentration, but less amounts of identified components present in *M. verticillata* seeds, it suggest that the inhibitory effect of WEMV might result from the combined effect of these chemical constituents such as polysaccharide, fatty acid or sterol in WEMV rather the action of individual constituents on osteoclast differentiation and resorption. Since we identified the presence of some chemical constituents as marker component for standardization of WEMV (data not shown), further studies to characterize the chemical properties of WEMV, to identify the unknown active components in WEMV, and to elucidate its action mechanism on osteoclast differentiation are needed to understand the effect of WEMV on osteoclasts and the action mechanism.

WEMV specifically inhibited the osteoclast differentiation potential of BMMs by inhibiting the RANKL signaling pathway. In TCM, *M. verticillata* seeds has been used to improve flow between the nutrient phase and the defense phase that enhances kidney function. Kidney has a function storing an essence of life, known as kidney essence, which generates and nourishes bone marrow to make a fullness of marrow in bone, forcefulness of musculoskeletal organ, and hardness of bone [14]. When kidney essence is exhausted by aging or disease, kidney function to provide the nutrition in bone marrow is insufficient to support bone microenvironment that deteriorates musculoskeletal function and increases pain in musculoskeletal organ, which is similar to manifestations observed in pathological bone diseases, such as osteoporosis and rheumatoid arthritis, caused by an imbalance of bone remodeling. Several studies have reported that herbs or herbal formulas originally used in TCM to tonify kidney function have positive effects on bone metabolism by directly inhibiting osteoclast differentiation [17] or resorption [34] or by regulating osteoblast-dependent osteoclastogenesis [35]. Regulation of the RANKL signaling on osteoclastogenesis has been suggested as one of the mechanisms by which herbal medicines tonifying kidney essence affect bone metabolism [36]. Thus, WEMV inhibition of the RANKL signaling pathway and bone resorption may be the molecular mechanism by which WEMV affects bone metabolism in addition to its pharmaceutic effect to strengthen kidney function in TCM.

## Conclusions

We have demonstrated that WEMV caused marked inhibition of osteoclastogenesis by suppressing the RANKL signaling axis and bone resorbing function by downregulating bone resorption markers. This study reveals the molecular mechanism underlying the effect of WEMV on osteoclastogenesis and suggests that WEMV may be a valid component of therapeutic prescriptions used in TCM to treat bone diseases caused by excess osteoclast differentiation and bone resorption.

## Abbreviations

ALP: Alkaline phosphatase
AP-1: Activator protein 1
BCA: Bicinchoninic acid assay
BMC: Bone marrow cell
BMM: Bone marrow macrophages
BMP-2: Bone morphogenetic protein-2
CA-NFATc1: Constitutively expressed form of NFATc1
CREB: cAMP response element binding
FBS: Fetal bovine serum
GC/MS: Gas chromatography/mass spectrometry
GFP: Green fluorescent protein
HPRT: Hypoxanthine phosphoribosyltransferase
IACUC: Institutional animal care and use committee
Id2: Inhibitor of DNA binding 2
JNK: c-Jun N-terminal kinase
MafB: v-Maf Avian musculoaponeurotic fibrosarcoma oncogene homolog B
MAPK: Mitogen-activated protein kinase
M-CSF: Macrophage colony stimulating factor
MNCs: Multinuclear osteoclasts
NFATc1: Nuclear factor of activated T-cells, cytoplasmic 1
NF-kB: Nuclear factor-kappaB
NIST: National Institute of Standards and Technology
PBS: Phosphate-buffered saline
PLC: Phospholipase C
PVDF: Polyvinylidene fluoride
qRT-PCR: Real-time quantitative polymerase chain reaction
RANKL: Receptor activator of nuclear factor-kB ligand
RIPA: Radioimmunoprecipitation assay
TCM: Traditional Chinese medicine
TRAP: Tartrate-resistant acid phosphatase
WEMV: Water extract of *M. verticillata* seeds.
